# IUC24415-81 Clinical outcomes in stage I seminoma patients aged ≥ 45 years according to treatment modality

**DOI:** 10.1093/oncolo/oyaf248.050

**Published:** 2025-08-29

**Authors:** F Mascaro, A Marmiroli, A Nuzzo, M Maruzzo, D Bimbatti, S Nazzani, M Claps, G Procopio, N Nicolai, P Giannatempo

**Affiliations:** Università degli Studi di Pavia, Pavia, Italy; Fondazione IRCCS Istituto Nazionale dei Tumori, Milano, Italy; Fondazione IRCCS Istituto Nazionale dei Tumori, Milano, Italy; Istituto Oncologico Veneto, Padova, Italy; Istituto Oncologico Veneto, Padova, Italy; Fondazione IRCCS Istituto Nazionale dei Tumori, Milano, Italy; Fondazione IRCCS Istituto Nazionale dei Tumori, Milano, Italy; Fondazione IRCCS Istituto Nazionale dei Tumori, Milano, Italy; Fondazione IRCCS Istituto Nazionale dei Tumori, Milano, Italy; Fondazione IRCCS Istituto Nazionale dei Tumori, Milano, Italy

## Abstract

**Background:**

Data regarding progression-free (PFS) and overall survival (OS) in clinical Stage I (CSI) seminomatous germ-cell tumors (SGCT) patients older than 45 years are limited. We address this knowledge gap, testing for these clinical outcomes according to treatment modalities within retrospective multicenter real-world data from 3 Italian institutions.

**Methods:**

Data from patients aged ≥ 45 years diagnosed with testicular CSI SGCT between 01/1992 and 07/2023 were collected. Descriptive analyses were performed and Kaplan–Meier curves were used to assess PFS and OS according to treatment modality, consisting of active surveillance [AS] vs adjuvant chemotherapy [CHT: 1 course of carboplatin AUC 7] vs adjuvant radiotherapy [RT: 20 to 30 Gy as paraortic strip or paraortic plus ipsilateral iliac nodes].

**Results:**

A total of 182 patients were selected. Of those, 120 (65%) underwent AS, 45 (25%) CHT, and 17 (10%) RT. Overall median age was 49.6 years (IQR 46.4-54.6). Patients undergoing AS were older (50.2 yo, IQR 47.2-55.3) than patients undergoing CHT (49.3 yo, IQR 46.4-51.3) or RT (49.3 yo, IQR 45.1-49.7). Most patients (98/182 or 54%) were aged between 45 and 50 years: 59 (60%), 27 (28%), and 12 (12%) underwent AS, CHT, and RT, respectively. Patients aged 51-65 were 74 (41%): 52 (70%), 18 (25%), and 4 (5%) underwent AS, CHT, and RT, respectively. Ten patients (5%) were aged >65: 9 underwent AS and 1 RT. (Table 1). After a median follow-up of 60 months, 5-year PFS was 83.7% for AS, 91.7% for CHT and 76.5% for RT (*P* = .41) (Figure 1). Relapses occurred in 18 (15%) vs 4 (9%) vs 5 (29%) patients in AS vs CHT vs RT, respectively. After a median follow-up of 60 months, 5-year OS was 100% across all treatment groups (*P* = .47).

**Conclusions:**

In CSI SGCT patients aged over 45, outcomes were excellent and aligned with the general CS I SGCT population, with 5-year PFS differences among treatment groups not reaching statistical significance. All relapsed patients were cured, and the 5yrs OS reached 100%. These results confirm the favorable prognosis of CSI SGCT even in older men.

**Figure 1.**
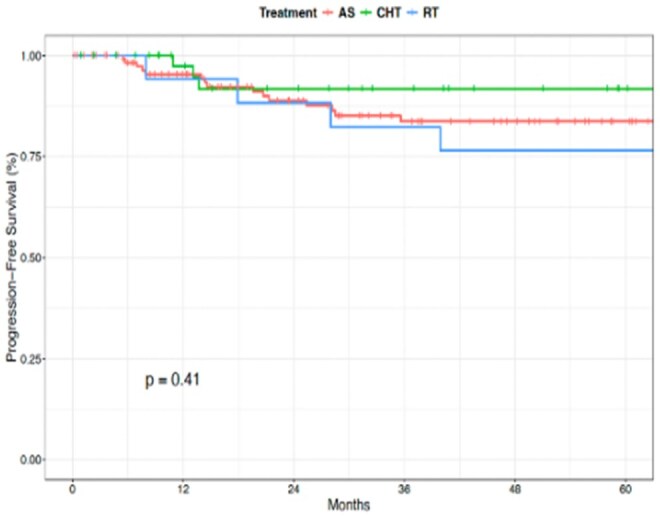
Kaplan-Meier curves depicting 5 years PFS in 182 CSI patients according to treatment modality.

**Table 1:** Age distribution of 182 Stage I seminoma patients according to treatment modality.

**Age groups (years)**	**Overall (*n*=182, 100%)**	**AS (*n* = 120, 66%)**	**CHT (*n* = 45, 25%)**	**RT (*n* = 17, 9%)**
45–50	98	59 (60%)	27 (28%)	12 (12%)
51–65	74	52 (70%)	18 (24%)	4 (6%)
≥66	10	9 (90%)	0	1 (10%)

